# Positive distraction in daily activities as a predictor of good coping: A “day in the life” during the COVID-19 pandemic

**DOI:** 10.3389/fpsyg.2023.1142665

**Published:** 2023-03-22

**Authors:** Calissa J. Leslie-Miller, Veronica T. Cole, Christian E. Waugh

**Affiliations:** ^1^Department of Psychology, William & Mary, Williamsburg, VA, United States; ^2^Department of Psychology, Wake Forest University, Winston-Salem, NC, United States

**Keywords:** stress, coping, COVID-19, positive distraction, multilevel modeling

## Abstract

**Introduction:**

The early part of the coronavirus pandemic (COVID-19) was a chronic stressor that led to decreased life satisfaction, increased psychopathology, and decreased social interaction, making it important to study coping strategies that stimulate increases in emotional well-being. Previous research has demonstrated that disengagement coping may be beneficial in scenarios where engagement coping is too difficult or not possible. We hypothesized that disengagement coping would be related to good emotional well-being (high positive emotions and/or perceived control, lower negative emotions and/or stress), with distraction (taking a break from a stressor) related to better emotional well-being than is avoidance (avoiding thoughts and feelings associated with a stressor).

**Methods:**

Using a daily reconstruction method that represents a “day in the life” of people in the United States during the early stages of the COVID-19 pandemic, we assessed people’s (N = 329) activities, their intention to distract from or avoid the stressor during these activities, emotions, and thoughts about and motivation to deal with COVID.

**Results:**

Between-subjects’ analyses revealed that habitual distraction did not predict any outcomes, while habitual avoidance related to poorer emotional well-being. Within-subject analyses, however, demonstrated that engaging in distraction (and to a smaller extent, avoidance) was associated with better concurrent emotional well-being and less thinking about COVID. Furthermore, the intent to distract/avoid was more reliable in predicting emotional outcomes than was the activity type.

**Conclusion:**

These findings suggest that disengagement from stress can be an adaptive coping behavior during global pandemics and possibly other chronic stressors with similar attributes.

## 1. Introduction

Very few Americans have experienced a shared stressor of comparable magnitude or duration to the COVID-19 pandemic. The early months of this novel chronic stressor led to decreased life satisfaction (Wanberg et al., 2020), increased psychopathology (Kujawa et al., 2020), and decreased social interaction (Hoffart et al., 2020). Although the COVID-19 pandemic affected people regardless of neighborhood, age, race, gender, or social economic status, it posed the greatest physical threat to older people (National Center for Health Statistics, 2020). Relative to younger adults, older adults were more susceptible to infection (Stokes et al., 2020), experienced worse symptoms (Garnier-Crussard et al., 2020), and were more likely to die due to illness (Wang et al., 2020). Additionally, they experienced substantial decreases in social interactions due to medical vulnerability (Garnier-Crussard et al., 2020), and therefore often experienced increased isolation, loneliness, and depression (Armitage and Nellums, 2020).

*Stressors*, such as the COVID-19 pandemic, are particular events that elicit stress responses including increased negative emotions (Feldman et al., 1999), decreased positive emotions (Ong et al., 2006), and sometimes differential physiological arousal (Selye, 1956). Stressor attributes are often used to help identify the consequences of the stressor on an individual, and include the controllability, the amount of uncertainty, the length of time, and the presence of other distractors. An individual’s response to stressors varies across individuals and within an individual from situation to situation. Individual coping strategy selection is associated with psychological well-being, such that use of maladaptive strategies is related to increased psychopathology (Aldao and Nolen-Hoeksema, 2012). Coping strategies are often categorized in a variety of ways, including as either engagement or disengagement strategies. Engagement coping strategies are those in which an individual directs energy at the stressor to manage the stress, while disengagement coping strategies are those in which an individual focuses away from the stressor to manage the stressor-related emotions (Moos and Schaefer, 1993; Carver and Connor-Smith, 2010). Engagement coping strategies include active coping, reappraisal, acceptance, and problem-solving, while disengagement coping strategies include distraction, avoidance, denial, and wishful thinking (Carver and Connor-Smith, 2010). For example, if an individual is laid off work, they may engage in the stressor through problem-solving by applying to new jobs, while they may disengage in the stressor through avoidance by going for a walk and ignoring the problem. Engagement coping strategies are widely regarded as beneficial. For example, reappraisal (Folkman and Moskowitz, 2000) and problem-solving (Moskowitz et al., 2009) are effective at reducing negative emotion.

While researchers have often highlighted the benefits of engagement strategies, they have typically cautioned against the use of disengagement coping strategies. This has led to engagement coping strategies being deemed constructive and disengagement coping strategies being identified as non-constructive strategies. However, disengagement coping research has shown mixed findings regarding its potential benefits with previous research, finding disengagement coping to be both ineffective (Brown et al., 2000; Langford et al., 2017), and effective for coping with stressors (Webb et al., 2012; Waugh et al., 2020). It is possible that these inconsistencies are a result of a lack of consideration for the attributes of the stressors. There may be stressors for which disengagement strategies are beneficial, such as in situations in which engaging with the stressor is not possible or too difficult or in stressors with high levels of long-lasting uncertainty and intensity (Waugh et al., 2020, 2021, 2022).

We suggest that disengagement coping strategies may be effective for dealing with the COVID-19 pandemic due to its stressor attributes: the high level of uncertainty, stressor duration, intensity, and presence of external distractors (i.e., any task that is not related to the stressor). The early months of the COVID-19 pandemic was a period of particularly high uncertainty, for a variety of reasons: the short-term and long-term consequences of illness progression, the likelihood of death to self or others, and the seemingly endless lockdown. Uncertainty makes it difficult to plan for the future (Miller, 1981), leading to both subjective and physiological experience of stress (Monat et al., 1972; de Berker et al., 2016). To reduce the stress associated with experiencing high levels of uncertainty, individuals attempt to find information to reduce the unknown (Berlyne, 1960). If this information is unavailable and individuals are unable to resolve the uncertainty, individuals may disengage from the stressor to decrease negative emotion (Miller, 1981). This may be particularly true for individuals experiencing stress associated with the COVID-19 pandemic, especially at the beginning when so little was known about the consequences or length of the pandemic.

Additionally, the COVID-19 pandemic was a widely experienced, physically and emotionally exhausting stressor of high intensity. Intense stressors are those that disrupt important activities (Larsen and Diener, 1987), are difficult to resolve (Martin and Tesser, 1996), and are long lasting (Sonnemans and Frijda, 1994; Waugh et al., 2010). When selecting coping strategies in the midst of intense stressors, disengagement strategies are often preferred (Sheppes et al., 2011). Miller (1981) suggested that when external distractors are present, disengagement strategy use increases. Early research on the COVID-19 pandemic has found that disengagement coping is highly used, above and beyond engagement coping strategies (Waugh et al., 2022). Social media usage, a common distractor, increased in usage during the pandemic (Conviva, 2020). It is possible that use of distraction was more easily accessible as a coping strategy during the COVID-19 pandemic, especially while individuals were in quarantine.

Our study is focused on avoidance and positive distraction, and acknowledges that these two disengagement strategies, although similar, are not equal (Waugh et al., 2020, 2021). While both involve focusing attention away from the stressor, positive distraction (i.e., taking a break from the stressor), has also been considered *engaging* in a distractor to temporarily relieve stress, with the intention to readdress the stressor later. While avoidance (i.e., the rejection of thoughts and behaviors related to the stressor), is solely *disengaging*, with no intention of returning to the stressor (Waugh et al., 2020). However, most of these prior findings on avoidance and distraction have assessed their habitual, or trait-like, use in dealing with all the stressors in one’s life. Instead, we predict that the effectiveness of disengagement coping may be best observed in the moment, as a temporary reprieve from COVID-19 related thoughts and worries, since disengagement coping has been found to be associated with short-term, but not long-term, well-being benefits (Waugh et al., 2022). Some evidence suggests that both distraction and avoidance are related to concurrent positive emotions, however, positive distraction, more consistently than avoidance, has been found to reduce negative emotions, disrupt rumination, and lead to more use of positive problem-focused coping (Skinner et al., 2003). Thus, distraction and avoidance might both be good in the moment, but the tendency to use distraction more so.

Previous research on the benefits of disengagement coping have focused on the self-restorative aspects (Kleiber et al., 2002), such that individuals can take the time to restore resources to be able to better engage in the stressor at a later time (Hamilton and Ingram, 2001; Skinner et al., 2003). Therefore, we also assessed people’s willingness and motivation to deal with the pandemic at each time point. We predict that after taking time to distract themselves from the COVID-19 pandemic, participants may be subsequently more motivated to engage in issues caused by the pandemic.

This present study aimed to demonstrate that disengagement coping strategies may be effective for dealing with the COVID-19 pandemic through daily diary. Participants reported on their daily activities, whether these activities were used for distraction or avoidance, and their well-being. We used multilevel modeling to determine the effect of participants’ momentary use of disengagement coping strategies for each episode of the daily diary. We hypothesized that momentary use of disengagement coping would be generally related to good contemporaneous emotional well-being (high positive emotions and/or perceived control, lower negative emotions and/or stress) and motivation to deal with COVID-19 related issues, with distraction potentially related to better emotional well-being than is avoidance.

## 2. Materials and methods

### 2.1. Participants

Participants were recruited in late March/early April of 2020, at the peak of the COVID-19 pandemic restrictions (U.S. Department of Defense, n.d.) using Qualtrics’ Panels in which potential participants have already agreed to be part of an online panel for sharing their thoughts and opinions for research. Qualtrics then identified those who were eligible (over 18 years old, reside in the US) and invited them to be part of the survey. Participants were 55.3% female and 88.4% white (M age = 58.27, SD age = 14.22). Given known drop-out rates with Qualtrics panels, we recruited enough initial participants (*N* = 1,499) to ensure that we would have at least 250 participants complete the parent study (Waugh et al., 2022), which consisted of three surveys and daily diaries. For this paper, we are focused on the daily diary portion of this study. The data analyses from this paper are not presented elsewhere. Although the initial recruitment goals were tailored for the sake of the parent study, a power simulation using the simr package in R (Green and MacLeod, 2016) indicated that the resulting sample size for this study (*N* = 329; see below) would be sufficient to detect the fixed effects of time-varying predictors in multilevel models. In a simulation with R = 1,000 replications, a small effect size for the predictor (b = 0.2) was detected 93.20% (95% CI = 91.46, 94.68) of the time when the ICC was set to 0.5.

### 2.2. Materials

Participants completed the daily diary entry at the end of the day – sometime after dinner but before bedtime. They were told that we were interested in what they did and how they felt that day. They were asked to reconstruct their day as if they were writing in a diary (i.e., Where were you? What did you do and experience? How did you feel? Kahneman et al., 2004). They listed each episode (up to 10) that occurred in the morning, afternoon, and evening and described what happened and what time it began and ended. An episode was included in the analyses if it included no more than one missing value for the ratings of that episode (M_episodes_ = 11.2, SD = 5.74). Participants were invited to complete up to 7 daily reconstruction method (DRM: Kahneman et al., 2004) daily diary entries. Unfortunately, participants did not complete many of these DRM entries with *n* = 434 completing 1, *n* = 68 completing 2, *n* = 16 completing 3, and *n* = 2 completing 4 (total *n* = 520). Because such a small percentage of the participants completed entries for more than 1 day, we analyzed data from everyone’s first complete DRM diary entry. This represents a ‘day in the life’ of people during the early part of the COVID-19 pandemic. After excluding everyone who did not have at least 1 complete DRM diary entry (at least 1 episode per time period: morning, afternoon, evening) the final DRM sample size was *n* = 329.

#### 2.2.1. Activity during episode

For each episode, participants indicated what activity they were doing during that episode including paid work, studying/schoolwork, commuting, shopping, housework/chores, eating/cooking, watching TV, reading, socializing, napping/resting, exercising, on computer, taking care of children, praying or meditating, grooming/self-care, errands, philanthropy, playing a game, on social media, something specifically related to dealing with the COVID-19 pandemic, other.

#### 2.2.2. Distraction/avoidance

For each episode, participants also indicated the nature of that episode as being “a pleasant activity to take a momentary break from thinking about or dealing with the coronavirus” [*distraction*], or “an opportunity to avoid thinking about or dealing with the coronavirus” [*avoidance*]. They also provided a number of other characterizations of the activity such as a personal success, a personal failure, a positive social interaction, a negative social interaction, a neutral social interaction, a thought/idea/realization, a goal was accomplished, a goal was blocked, being free from thought, caught up in the moment, a reaction to something I saw or heard, and other. Participants were able to select all characterizations that applied. These other descriptions are beyond the scope of this article and will not be analyzed, however, researchers interested in these data are invited to contact us.

#### 2.2.3. Well-being during episode

For each episode, participants rated aspects of their well-being during that episode on a 0 (not at all) to 6 (very) scale. They reported on how *stressed, in control of feelings, pleasant* (positive emotions), *and unpleasant* (negative emotions) they felt during the episode. These single-item scales are treated separately in the analyses.

#### 2.2.4. COVID-19 related thoughts and motivation

For each episode, participants reported on how often they thought about the COVID-19 from 0 (not at all) to 6 (very) and how motivated they would be to engage in some activity related to dealing with issues caused by the COVID-19 pandemic after this episode from 1 (not motivated at all) to 4 (very motivated).

#### 2.2.5. Future thinking

Lastly, participants reported on how often during that episode they thought about a future positive/negative/neutral event activity or goal from 1 (not at all) to 4 (very often). These data are not presented here, but can be found in a separate manuscript (Leslie-Miller et al., 2021).

### 2.3. Data analysis

To evaluate whether disengagement coping would be related to good emotional well-being, we conducted separate multilevel models with episode distraction and episode avoidance (dummy-coded as 0 or 1 according to whether or not they endorsed it for each episode) as predictors of aspects of emotional well-being. For each of these models, episodes were the Level 1 unit nested within participant at Level 2. In each model, the focal predictor was the within-person component, which was calculated by centering around the participant’s mean (here the proportion of times each participant used either distraction or avoidance). The between-person component (i.e., person-level mean) was also included in each model. By centering in this way, within-person and between-person variance components are orthogonal and therefore can be included in the models together (Raudenbush and Bryk, 2002), which allowed us to separate between-subjects relationships (i.e., those who tended to use distraction/avoidance and how they generally felt) from within-subjects relationships (i.e., how did using distraction/avoidance during a given episode relate to well-being outcomes for that episode). On some models, we also included distraction and avoidance together to assess whether the effects of each persisted when controlling for the other. Therefore, in total we ran 18 models (3 predictor sets: distraction, avoidance, distraction + avoidance × 6 dependent variables: positive emotions, negative emotions, stress, control of feelings, thinking about COVID, and motivation to deal with COVID). We corrected for multiple comparisons separately for between-and within-subjects effects using a Benjamini-Hochberg correction (Benjamini and Hochberg, 1995). Given that the combined model (i.e., distraction + avoidance) is not independent of the separate models for distraction and avoidance, we defined the number of independent tests as 12.

We also examined whether any of the activities predicted within-person changes in well-being outcomes. We again conducted MLM models but this time each separate model included the activity (dummy-coded as 0 or 1 and then person-centered) and person-level activity mean as predictors and each aspect of emotional well-being as a dependent variable. In total, we ran 120 models (20 predictors: computer, TV, dining, exercise, social media, playing game, reading, socializing, praying/meditating, caring for child, napping/resting, shopping, housework, something related to COVID, self-care/grooming, errands, studying, paid work, philanthropy, commuting x 6 dependent variables: positive emotions, negative emotions, stress, control of feelings, thinking about COVID, and motivation to deal with COVID). We again controlled for multiple comparisons using a Benjamini-Hochberg correction, this time defining the number of tests as 120.

## 3. Results

### 3.1. Descriptives

**Figure 1:**
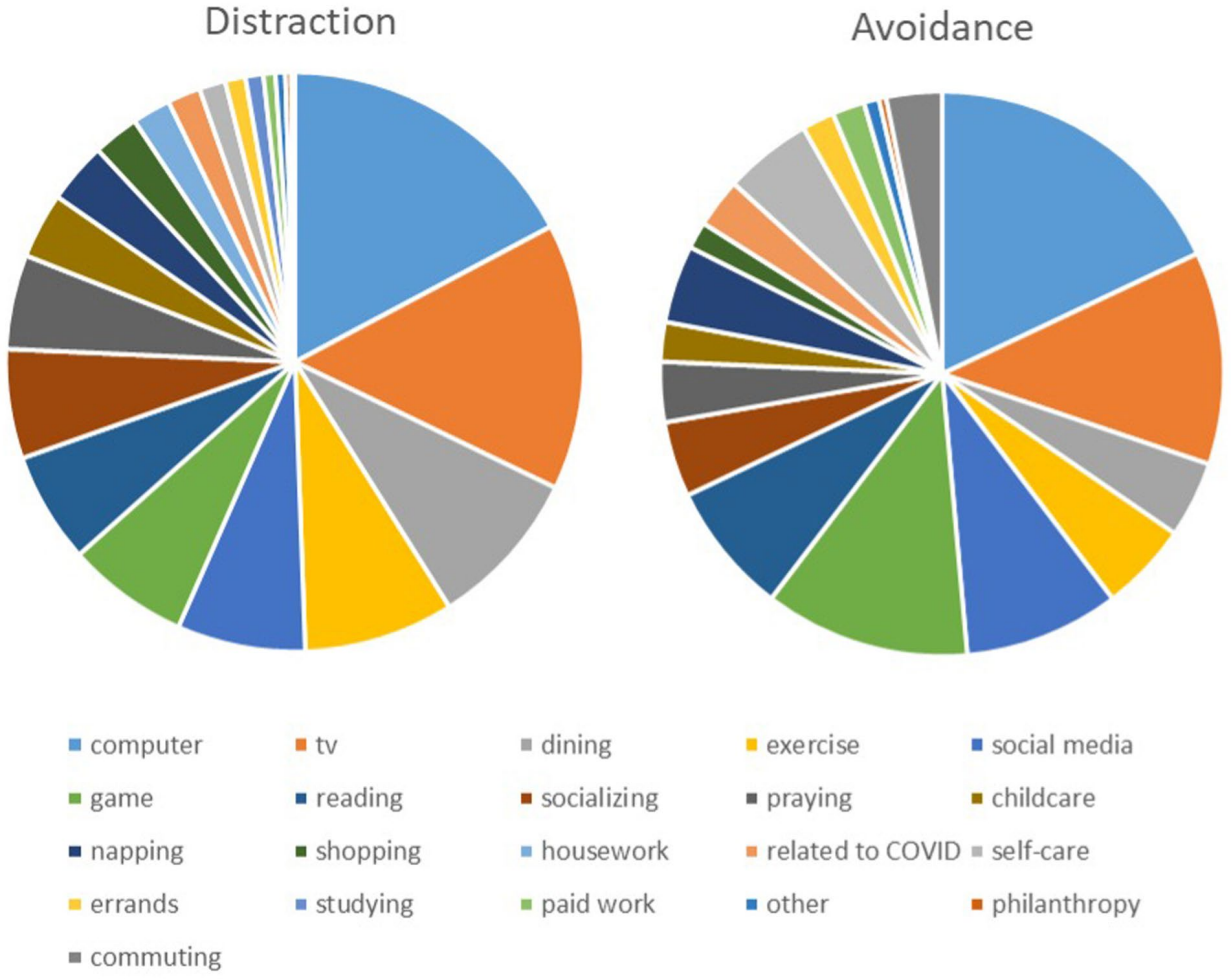
Relative frequencies of actives used for distraction and avoidance as reported in the daily diaries.

On average, people distracted themselves on 14.7% of their episodes and used avoidance on 6.5%. Participants distracted themselves the most by using the computer, watching TV, dining, exercising, and using social media (Figure 1). Participants avoided thinking about the COVID-19 pandemic the most by using the computer, watching TV, playing a game, using social media, and reading (Figure 1).

### 3.2. Multilevel modeling of the relationship between distraction/avoidance and well-being during daily events

#### 3.2.1. Between-subject relationships

Individual differences in habitual distraction use did not predict any of the well-being outcomes. People who tended to habitually use more avoidance showed higher negative emotions, stress and thinking about COVID.

#### 3.2.2. Within-subject relationships

When participants used distraction during an activity, it predicted higher positive emotions and control, and lower negative emotions, stress and thinking about COVID during that activity. This pattern of findings was the same with avoidance activities as well, albeit fairly weaker and without a positive effect on control (Table 1). Importantly, these effects for each strategy persisted when controlling for the other strategy.

**Table 1:** Multilevel models of the relationship between distraction, avoidance, activities and emotional responses during daily diary events.

	Between-subject						Within-subject: X predicting Y					
X↓ / Y→	PE	NE	Str	CTL	Think COVID	Motiv COVID	PE	NE	Str	CTL	Think COVID	Motiv COVID
Distraction	−0.08	0.04	0.04	−0.05	0.08	−0.03	0.15**	−0.1**	−0.1**	0.06**	−0.07**	−0.01
Distraction (ctrl Avoidance)	−0.05	−0.07	−0.08	0.00	−0.07	−0.08	0.14**	−0.10**	−0.09**	0.06**	−0.07**	−0.01
Avoidance	−0.07	0.17**	0.2**	−0.09	0.17**	0.06	0.06**	−0.04**	−0.06**	0.03*	−0.07**	−0.01
Avoidance (ctrl Distraction)	−0.07	0.23**	0.26**	−0.10	0.19**	0.11	0.04**	−0.04*	−0.05**	0.02	−0.07**	−0.01
	Activities											
Computer	−0.03	0.06	0.03	0	0.03	−0.1	0	0.03	0.03	0.01	0.02	−0.01
TV	0.05	0.19**	0.17*	0.02	0.1	−0.02	0	0.01	0.01	0.01	0.07**	0
Dining	0.01	0.14**	0.14**	−0.01	0.11*	−0.03	0.02	0.01	−0.01	0	0.04	0.02
Exercising	−0.04	0.15**	0.16**	−0.08	0.11*	−0.04	0.02	−0.01	−0.01	0.03	−0.01	0.02
Social Media	−0.03	0.18*	0.2**	−0.07	0.18**	0.08	0.01	0.01	0	0.01	0.04	−0.02
Playing Game	−0.03	0.15	0.13	−0.02	0.12	0.07	0.03	0.01	−0.01	0.02	−0.02	0
Reading	0.02	0.26**	0.26**	0	0.18**	−0.05	−0.01	0.01	0.01	0	0.03	0
Socializing	0.08	0.07	0.08	0.04	0.08	0.09	0.02	−0.02	−0.02	0.01	0	−0.02
Praying/Meditating	0.04	0.11	0.14*	−0.01	0.05	0.01	0.02	0	−0.01	0.02	−0.01	0.03
Caring for child	0.05	0.06	0.09	0.02	0.07	0.04	0.01	−0.01	−0.01	0.01	−0.01	0
Napping/Resting	−0.07	0.04	0.05	−0.1	0.02	−0.03	0.01	0.01	−0.01	−0.01	−0.01	−0.03
Shopping	0.07	0.29**	0.3**	0	0.19**	0.04	−0.01	0.02	0.02	−0.01	0.04	−0.01
Housework	0.04	0.09	0.1*	0.05	0.07	0.07	0	−0.01	0.01	−0.01	0.01	0.01
Something related to COVID	0	0.01	0.01	−0.02	0.04	−0.03	−0.02	0.05**	0.06**	0	0.06**	0.01
Self-care/grooming	0.03	0.02	0.02	0.05	0.01	0.04	0.01	−0.01	−0.03	0.01	−0.01	0.01
Errands	0.07	0.05	0.06	0.05	0.04	0.07	−0.03	0.02	0.03	0	0.06**	0.02
Studying	0.04	0.09	0.1	0	0.04	0.01	−0.01	0	0	0	0	0
Paid work	−0.02	0.15**	0.17**	−0.02	0.11**	0	0.03	0	−0.01	0	0	0.01
Philanthropy	0.07	0.26**	0.29**	0.05	0.18	0.1	0	0	0.01	0	0	0
Commuting	0.03	0.12**	0.15**	0.01	0.12*	0.08	0.01	0.02	−0.01	0	0.02	−0.01

Activities are shown in descending order of the frequency with which people use them to distract themselves from COVID. Standardized betas are shown. PE = positive emotion, NE = negative emotion, Str = stress, CTL = control, motiv = motivation.

** *p* < 0.05.

* *p* < 0.01.

#### 3.2.3. Activities

Although there were a couple of relationships (e.g., perhaps unsurprisingly, watching TV was associated with thinking more about COVID), none of the activities showed the same pattern or strength of relationships with well-being outcomes as were shown with distraction and avoidance (Table 1).

## 4. Discussion

The purpose of the present study was to test whether disengagement coping could be an effective coping strategy for a novel chronic stressor, the COVID-19 pandemic. We found that distraction was used fairly frequently, with the most popular distraction activities consisting of computer use, watching TV, dining, exercising, and using social media. As anticipated, social media was a common and easily accessible distraction (Conviva, 2020), which was particularly suitable for the COVID-19 pandemic. This finding supports the previous research that stressors with high uncertainty, long lasting intensity, and external distractors encourages the use of distraction strategies (Miller, 1981). However, we do not have pre-pandemic levels, which means that although distraction seemed to be a common reason for choosing activities (14.7% of their episodes on average), we cannot determine whether it was used more often during the pandemic than before the pandemic.

Consistent with our hypotheses, in addition to being selected at a relatively high rate, distraction was related to emotional well-being outcomes in the moment. Relative to when they engaged in non-distraction activities, when people engaged in distraction activities, they reported higher positive emotions and control, and lower negative emotions, stress, and thinking about COVID. Interestingly, similar, though weaker, patterns were found for when people used activities as an avoidance strategy; during those activities they demonstrated higher negative emotions and stress, and less positive emotions. On a between-subject level, habitual use of distraction did not predict individual differences in any of the outcomes, whereas those higher in habitual avoidance exhibited more negative emotions and stress. These findings suggests that within-subject analyses may better account for the relationship between momentary use of disengagement coping and well-being and add to the literature exploring the similarities and distinctions between distraction and avoidance (Waugh et al., 2020).

These findings are consistent with previous research that has focused on the self-restorative benefits of disengagement coping (Kleiber et al., 2002). Consistent with prior research (Waugh et al., 2022), we found that disengagement coping is beneficial as a temporary reprieve. We add to that literature to suggest that this temporary reprieve can be really captured in moment to moment (i.e., minutes to hours) changes in emotional well-being. Similarly to Skinner et al. (2003), we found that positive distraction reduced negative emotions, and we support the literature that has found disengagement coping to be effective for coping with stressors (Webb et al., 2012; Waugh et al., 2020). However, this conflicts with previous research that has found disengagement coping to be highly associated with psychological distress (Brown et al., 2000; Langford et al., 2017).

There are many possible explanations for the inconsistencies found in research on disengagement coping. We hypothesized that these inconsistencies are a result of a lack of consideration for the attributes of the stressors, which is consistent with the strategy-situation-fit coping flexibility literature that highlights the match between the demands of the stressor and the coping strategy type (Cheng et al., 2014). High uncertainty, longevity, and the presence of external distractors may be key stressor attributes when determining stressors in which disengagement coping would be beneficial. Alternatively, it is possible that study design has influenced these discrepancies, since within-person and between-person designs can produce extremely different, even opposite, results (Curran and Bauer, 2011).

Notably, participants used very similar activities to either distract themselves or avoid thinking about COVID. We found some relationships between activities and well-being (e.g., perhaps unsurprisingly, watching TV was associated with thinking more about COVID), but none demonstrated the same pattern or strength of relationships with emotional outcomes as did distraction or avoidance. Indeed, some of the activities that were the most popular while distracting/avoiding showed opposite relationships with negative emotions (e.g., COVID-related activities) and thinking about COVID (e.g., watching TV) than did the intent to distract or avoid. This suggests that the intention to distract/avoid may be more important in predicting well-being than is the type of activity.

Importantly, these findings are specific to the early period of the COVID-19 pandemic with high levels of uncertainty, longevity, and the presence of external distractors. As these characteristics change throughout the pandemic, it is possible that the adaptive value of disengagement coping as well as other coping strategies may change as well. Importantly, however, although the focus in this paper was on a global pandemic, these findings may also be relevant when addressing other stressors with these key attributes (high levels of uncertainty, longevity, and the presence of external distractors), such as caregiving for a child with a chronic illness (Waugh et al., 2021). Future research is needed to explore the value of disengagement strategies across varying situations, cultures, and ages. Our study was limited to individuals in the United States during the COVID-19 pandemic and therefore, cannot generalize to individuals worldwide. Additionally, given that the COVID-19 pandemic posed the greatest physical threat to older people (National Center for Health Statistics, 2020), it is important to note that our recruitment method unintentionally resulted in an older adult sample. Therefore, we do not know if our findings would be supported in a younger sample. Similarly, our sample is majority white, therefore, our results may not generalize to non-white samples and future research should seek to explore the role of disengagement coping in more diverse samples.

Importantly, given the cross-sectional nature of this study, we were unable to determine the directionality of these effects leaving open the very real possibility that the relationship between coping and emotional well-being is bidirectional. Additionally, because participants rated the aspects of the episodes retrospectively, it’s possible that certain terms used in the scale items (like ‘pleasant’ when describing distraction activities) primed the participants to then think of these activities as inducing positive emotion. Although this priming effect does not parsimoniously explain the relationships between avoidance and emotional outcomes, future studies should use ecological momentary assessments throughout the day and counterbalance statements to address these limitations. Importantly, we chose to use single-item questions because it reduces respondent burden (Fisher et al., 2015) in studies where participants are making many ratings of many episodes throughout a day. We have successfully used these single-item questions many times before (Waugh et al., 2020; Leslie-Miller et al., 2021; Waugh et al., 2021) and there are several examples of studies demonstrating the validity and reliability of single-item scales especially when it comes to emotional outcomes (e.g., Wanous and Hudy, 2001; Abdel-Khalek, 2006). Despite our limitations, our findings add valuable support for the literature demonstrating that disengagement is effective for producing momentary reprieves from intense chronic stressors. These findings suggest that distraction can be an adaptive coping behavior during global pandemics and potentially additional stressors with similar attributes.

## Data availability statement

The datasets presented in this study can be found in online repositories. The names of the repository/repositories and accession number(s) can be found in the article/supplementary material.

## Ethics statement

The studies involving human participants were reviewed and approved by The Wake Forest University Institutional Review Board. The patients/participants provided their written informed consent to participate in this study.

## Author contributions

CL-M, CW, and VC were involved in the design of the study. CL-M and CW were involved in conducting the study. CW and VC were involved in data analysis. All authors contributed to the article and approved the submitted version.

## Funding

This project was funded by a Wake Forest University Collaborative Pilot Grant to CW.

## Conflict of interest

The authors declare that the research was conducted in the absence of any commercial or financial relationships that could be construed as a potential conflict of interest.

## Publisher’s note

All claims expressed in this article are solely those of the authors and do not necessarily represent those of their affiliated organizations, or those of the publisher, the editors and the reviewers. Any product that may be evaluated in this article, or claim that may be made by its manufacturer, is not guaranteed or endorsed by the publisher.
